# MUC16 Retention after Neoadjuvant Chemotherapy in Pancreatic Ductal Adenocarcinoma

**DOI:** 10.3390/cancers16203439

**Published:** 2024-10-10

**Authors:** Kathryn M. Muilenburg, Evie G. Ehrhorn, Madeline T. Olson, Carly C. Isder, Kelsey A. Klute, Geoffrey A. Talmon, Mark A. Carlson, Quan P. Ly, Aaron M. Mohs

**Affiliations:** 1 Department of Pharmaceutical Sciences, University of Nebraska Medical Center, 505 S 45 St, Omaha, NE 68198, USA; kathryn.muilenburg@unmc.edu (K.M.M.); carlyisder@outlook.com (C.C.I.); 2 Fred and Pamela Buffett Cancer Center, University of Nebraska Medical Center, 505 S 45 St, Omaha, NE 68198, USA; eehrhorn@unmc.edu (E.G.E.); maddieolson19@gmail.com (M.T.O.); kelsey.klute@unmc.edu (K.A.K.); gtalmon@unmc.edu (G.A.T.); macarlso@unmc.edu (M.A.C.); qly@unmc.edu (Q.P.L.); 3 Eppley Institute for Research in Cancer and Allied Diseases, University of Nebraska Medical Center, 505 S 45 St, Omaha, NE 68198, USA; 4 Department of Internal Medicine, University of Nebraska Medical Center, 42nd and Emile, Omaha, NE 68198, USA; 5 Department of Pathology and Microbiology, University of Nebraska Medical Center, 985900 Nebraska Medical Center, Omaha, NE 68198, USA; 6 Department of Surgery, University of Nebraska Medical Center, 983280 Nebraska Medical Center, Omaha, NE 68198, USA; 7 Department of Biochemistry and Molecular Biology, University of Nebraska Medical Center, S 45th St, Omaha, NE 68198, USA

**Keywords:** fluorescence-guided surgery, MUC16, pancreatic ductal adenocarcinoma, neoadjuvant therapy, protein expression

## Abstract

**Simple Summary:**

Neoadjuvant chemotherapy has risen in clinical use for the treatment of pancreatic cancer over the past few decades. Many patients now undergo prior treatment before surgical resection to aid in downstaging the tumor for a potentially improved surgical outcome. Potential targeted therapies such as fluorescence-guided surgery often rely on the upregulated protein expression in the tumor. Therefore, it is important to understand the impact of neoadjuvant therapy on tumor protein expression, as protein expression may be altered by chemotherapy treatment. The aim of our study was to evaluate the expression of Mucin 16 (MUC16) after neoadjuvant chemotherapy in pancreatic ductal adenocarcinoma tissue. This study found that MUC16 expression was retained in the tumor following neoadjuvant chemotherapy treatment regimens. These findings may have a broad impact on the delivery of MUC16-targeted therapies before and after neoadjuvant chemotherapy treatment.

**Abstract:**

**Background/Objectives**: Pancreatic ductal adenocarcinoma (PDAC) has a poor prognosis. Currently, surgical resection is the only potentially curative treatment. Unfortunately, less than 20% of PDAC patients are eligible for surgical resection at diagnosis. In the past few decades, neoadjuvant chemotherapy treatment (NCT) has been investigated as a way to downstage PDAC tumors for surgical resection. Fluorescence-guided surgery (FGS) is a technique that can aid in increasing complete resection rates by enhancing the tumor through passive or active targeting of a contrast agent. In active targeting, a probe (e.g., antibody) binds a protein differentially upregulated in the tumor compared to normal tissue. Mucin 16 (MUC16), a transmembrane glycoprotein, has recently been explored as an FGS target in preclinical tumor models. However, the impact of chemotherapy on MUC16 expression is unknown. **Methods**: To investigate this issue, immunohistochemistry was performed on PDAC patient samples. Results: We found that MUC16 expression was retained after NCT in patient samples (mean expression = 5.7) with minimal change in expression between the matched diagnostic (mean expression = 3.66) and PDAC NCT patient samples (mean expression = 4.5). **Conclusions**: This study suggests that MUC16 is a promising target for FGS and other targeted therapies in PDAC patients treated with NCT.

## 1. Introduction

Pancreatic cancer is expected to be the 2nd most lethal cancer by 2030 [1]. The current standard of care for pancreatic cancer consists of surgical resection, chemotherapy, and, less commonly, chemoradiation therapy, depending on the tumor stage [2,3]. However, surgical resection remains the only potentially curative treatment for pancreatic cancer [4,5]. Surgeons aim to achieve a complete tumor resection to increase the patient’s overall survival [6]. Preoperative imaging such as PET and MRI allow a surgeon to visualize the tumor in a static environment. However, the intraoperative field is dynamic, wherein surgeons must rely on visual and tactile clues to resect the tumor [7]. Due to the lack of intraoperative imaging tools, tumor infiltration, spread to lymph nodes, high stromal content, and surgical complexity, incomplete resections occur in up to 60% of surgically resected pancreatic cancer patients within 3 years of resection, resulting in a decreased 5-year survival [8,9,10].

To improve the rate of complete resections, researchers are focusing on developing intraoperative tools like fluorescence-guided surgery (FGS) to aid in surgical resection. FGS utilizes a fluorescent dye or dye conjugate (e.g., antibody) to target differentially and fluorescence a tumor compared to the surrounding normal tissue [6,11]. Currently, potential FGS applications are limited in pancreatic cancer, as less than 20% of patients are diagnosed with resectable disease; however, another 30% of PDAC patients present with borderline resectable or locally advanced disease [4,12,13]. While not eligible for resection at diagnosis, these patients may be eligible for surgery after neoadjuvant therapy if there is a sufficient reduction in tumor burden [14]. Neoadjuvant chemotherapy treatment (NCT) is a standard pre-surgery regimen, often consisting of a FOLFIRINOX or gemcitabine-based chemotherapy regimen. The clinical use of NCT has been trending up over the last two decades due to clinical research pinpointing the regimen’s ability to downstage the tumor and improve overall survival [2,4,12,14,15]. Current research has shown that neoadjuvant FOLFIRINOX or gemcitabine and capecitabine increases post-surgical survival compared to surgery alone [12].

NCT can lead to aberrant protein expression in treated tumors compared to untreated tumors [16,17]. Protein retention after chemotherapy treatment is vital for FGS and other targeted therapies, including CAR T-cell therapy, antibody-drug conjugates, nanoparticles, and follow-up chemotherapy treatment. Several proteins, such as Mucin 16 (MUC16), have been evaluated as FGS targets in pancreatic ductal adenocarcinoma (PDAC), the most common type of pancreatic cancer [5,18,19,20,21,22,23,24]. Additionally, proteins such as CEA, EGFR, VEGF-A, and integrins have been studied or are under study as targets for FGS of pancreatic cancer in clinical trials [21,25,26,27,28]. Targeted FGS has also shown promise in other tumors. Within the past 5 years, the FDA has approved pafolacianine (OTL38) for FGS in ovarian and lung cancers and LUM015 for FGS in breast cancer [29,30,31]. MUC16, a mucin family member, is a transmembrane protein upregulated in 60–80% of PDAC patients [32,33]. Specifically, MUC16 has been investigated as a target for FGS in PDAC using the MUC16-targeting antibody, AR9.6, and its humanized form, huAR9.6 [5,20]. In addition to PDAC, MUC16 upregulation is found in many other tumors [22]. As NCT becomes the standard treatment for PDAC and other cancers, it is imperative to determine if MUC16 is durably expressed and, thus, a potential FGS target candidate.

To investigate MUC16 expression after NCT in PDAC patients, we obtained samples that did (NCT) or did not receive NCT prior to surgical resection (non-NCT). We hypothesized that MUC16 expression would be unchanged in samples that received chemotherapy treatment before resection compared to samples that did not receive treatment before resection. Here, we evaluated the expression of MUC16 following NCT using banked samples from PDAC patients. Our findings indicate that MUC16 is expressed after NCT in PDAC patient specimens.

## 2. Methods and Materials

### 2.1. Ethics

All de-identified samples in this study were selected from a tissue bank at the University of Nebraska Medical Center (UNMC) and from the Department of Pathology (UNMC). Written informed patient consent and UNMC Institutional Review Board (IRB) approval were obtained for all samples in the tissue bank prior to the banking of the samples. As per the United States Department of Health and Human Services regulations policy, 45 CFR 46.102(e), this study did not constitute human subjects research because the samples were not originally banked for this research, a third-party honest broker selected the samples, and no identifiable information was gathered or known by any of the co-authors. Due to Policy 45 CFR 46.102(e), specific written informed consent and IRB approval were not required for the deidentified samples obtained through a third-party individual from the tissue bank and Department of Pathology (UNMC). This research was conducted in accordance with the UNMC Institutional Review Board guidelines, the United States Department of Health and Human Services regulations, and the Declaration of Helsinki.

### 2.2. Acquisition of Normal Pancreatic Tissue

Pancreatic cancer most commonly develops in the head of the pancreas [34]. Therefore, samples were selected from tumors derived from this pancreas region. Formalin-fixed paraffin-embedded (FFPE) normal pancreatic head patient samples (*N* = 10) were obtained from the Normal Organ Recovery (NORs) program, a subset of the Rapid Autopsy Program (RAP), UNMC (IRB #091-01).

### 2.3. Acquisition of Pancreatic Ductal Adenocarcinoma Patient Sample Slides

FFPE PDAC specimens were obtained from the Paraffin Tissue Bank, UNMC, and the Department of Pathology, UNMC, through an honest broker. Patient specimens were obtained according to the status of NCT and surgical resection (36 NCT specimens, 17 exploratory pre-treatment biopsy or cytology (diagnostic) samples of NCT specimens, 32 non-NCT specimens, and 60 NCT and non-NCT matched adjacent specimens). The specimens were separated into four groups—NCT (*N* = 35), diagnostic (*N* = 16), non-NCT (*N* = 31), and matched adjacent tissue (*N* = 57, *N* = 28 for non-NCT, and *N* = 29 for NCT) according to the inclusion criteria. Two diagnostic specimens had two separate blocks available. Samples were received from both blocks, and each sample’s mean (M) expression was reported. For NCT samples, the standard-of-care chemotherapy regimen was defined as a FOLFIRINOX or gemcitabine-based chemotherapy treatment regimen [12,13]. Any non-FFPE samples or tumor-free NCT or non-NCT tissues were excluded from the study. Any matched diagnostic sample without atypical tissue or tumor was excluded from the study. Finally, any adjacent tissue without a matched NCT or non-NCT sample was excluded from the study. Pathologic stage designations for primary tumor, lymph node, and metastatic disease were obtained for each sample and defined according to the 8th edition of the American Joint Committee on Cancer (AJCC) for pancreatic cancer [35,36,37,38]. Primary tumor designations were defined as T1: tumor less than or equal to 2 cm, T2: tumor greater than 2 cm but less than 4 cm, T3: tumor greater than 4 cm, and T4: tumor extends to and involves pancreatic vasculature and arteries. The NCT sample primary tumor designations were reclassified after chemotherapy treatment. Lymph node designations were defined as N0: no affected lymph nodes, N1: 1–3 affected regional lymph nodes, and N2: greater than 4 affected regional lymph nodes. Metastatic disease designations were defined as MX: unknown metastasis, M0: no distant metastasis, and M1: distant metastatic disease is present. All tumors were staged according to the modified 8th edition of the AJCC for pancreatic cancer [35,36,37,38].

### 2.4. Immunohistochemistry and Hematoxylin and Eosin Staining of Samples

All samples were stained with the OC125 (CA125) mouse monoclonal antibody (Roche Diagnostics, 760-2610, Manufacturer: Cell Marque Corporation, Rocklin, CA, USA) by the Tissue Science Facility, UNMC. The antigens were retrieved in Cell Conditioning 1 (CC1) buffer at 95 °C for 64 min. The primary antibody, OC125, was incubated at 37 °C for 24 min. The secondary antibody, Ventana OptiView horseradish peroxidase antibody (Roche Diagnostics, 760-700, Manufacturer: Ventana Medical Systems, Inc., Tucson, AZ, USA), was incubated for 12 min. Slides were counterstained with hematoxylin.

PDAC, matched adjacent, and diagnostic samples were stained for tissue structure with hematoxylin and eosin staining by the Tissue Science Facility, UNMC. The samples were deparaffinized and rehydrated using xylenes and a gradient of ethanol and water. The hematoxylin and eosin kit, Select Hematoxylin and Reserve Eosin Multichrome (StatLab Medical Products, SL401, and SL201, McKinney, TX, USA), was used to stain the slides in the Tissue-Tek Prisma Automated Slide Stainer (Sakura Finetek, Torrance, CA, USA) according to the manufacturer’s protocol.

The IHC and H&E slides were imaged on the Olympus IX73 microscope with a DP80 camera (Evident, Olympus Life Science, Tokyo, Japan) at 20× magnification in the brightfield channel using the cellSens Dimension software version 1.18 (Evident, Olympus Life Science, Tokyo, Japan).

### 2.5. Sample Analysis by Pathologist

All slides were analyzed by a board-certified pathologist blinded to the tissue type and treatment status. The specimens were analyzed for the tissue type, tumor grade, fibrosis classification, and pathological response grade. The fibrosis classification was designated by the amount of fibrotic tissue in the section. The pathological response was graded on a scale of minimal to maximum response. Both the fibrosis classification and grade of pathological response were graded to the best of the pathologist’s ability according to the tissue structure on the H&E-stained slides. The extent of MUC16 expression was analyzed through stain intensity, the percentage of tumor cells stained, and the location of the stain.

MUC16 stain intensity was graded on a 0–3 scale (0, 1+, 2+, 3+) [7,32,39,40,41]. The percentage of cells stained was classified on a 0–4 scale (0—no positive cells, 1—under 25%, 2—26–50%, 3—51–75%, and 4—greater than 75%) [7,32]. The immunoreactive score (IRS) was calculated by multiplying the stain intensity by the percentage of cells stained [7,32,40,41]. The IRS scores ranged from 0–12. The assigned scoring was as follows: 0–1—no expression, 2–3—mild expression, 4–8—medium expression, and 9–12—high expression [7,40,41].

### 2.6. Statistical Analysis

All analyses were completed in GraphPad Prism versions 9 and 10 (GraphPad Software, La Jolla, CA, USA). Prior to the start of the study, a power analysis was performed to determine the number of samples needed for statistical significance. With an alpha of 0.5 and a power of 80%, it was determined that a range of 22 to 35 samples was needed to determine statistical significance. MUC16 expression was analyzed using a two-tailed Mann–Whitney test for unpaired samples with a non-Gaussian distribution [32,39,42]. A two-tailed Wilcoxon test for paired samples was used to analyze matched tumor and adjacent tissue samples. *p* < 0.05 was considered to be significant.

## 3. Results

### 3.1. Patient Sample Characteristics

To investigate the impact of NCT on MUC16 expression, 66 PDAC patient samples were obtained for this study. Thirty-one (47.0%) patients were classified as non-NCT. Thirty-five (53.0%) patients received NCT. The sample characteristics can be found in Table 1.

### 3.2. MUC16 Expression in PDAC Patient Samples

**Figure 1:**
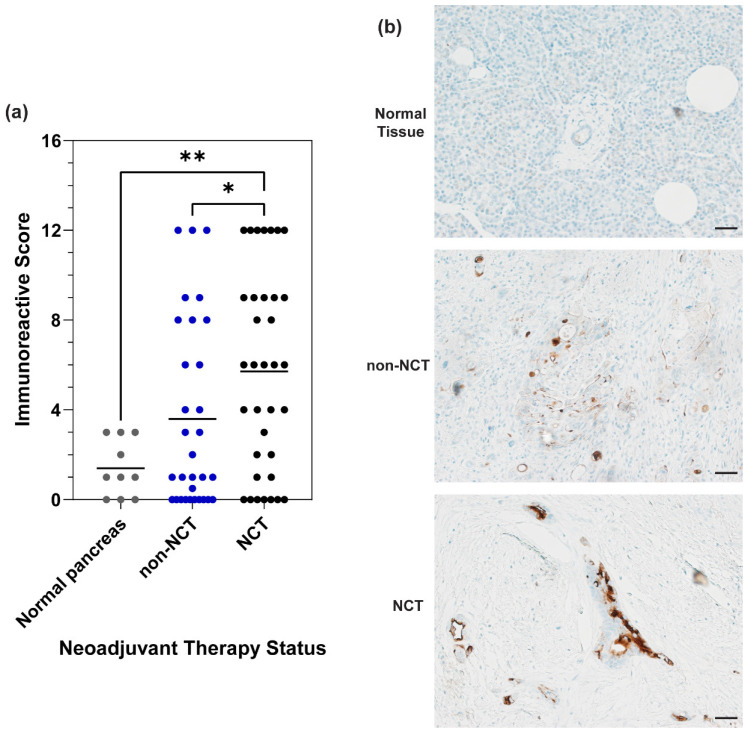
Mucin 16 (MUC16) is expressed in both NCT and non-NCT patient samples. (**a**) Dot plot with all samples comparing the mean immunoreactive score (IRS) of the NCT, non-NCT, and normal pancreas specimens. Data were analyzed using the Mann–Whitney test. *p* < 0.05; * 0.01 < *p* < 0.05; ** 0.001 < *p* < 0.01. (**b**) Representative images of the mean MUC16 expression in each group were taken at 20× magnification. Scale bar = 50 µm.

MUC16 expression was retained in PDAC patient specimens following NCT (Figure 1a,b). NCT specimens had a statistically significant higher MUC16 expression (M = 5.71, *SD* (standard deviation) = 4.41) compared to the normal pancreas (M = 1.40; *SD* = 1.26) (*p* = 0.0066) and non-NCT (M = 3.60, *SD* = 4.15) (*p* = 0.0494) specimens (Figure 1a). However, there was not a statistically significant difference in MUC16 expression between the normal pancreas and non-NCT specimens (*p* = 0.3785). Within the NCT sample set, MUC16 was expressed in tissues treated with a FOLFIRINOX-based regimen or a gemcitabine-based regimen (Figure S1a). Additionally, 48.6% (17/35) of the NCT samples received some radiation treatment following the chemotherapy regimen. The NCT samples expressed MUC16 at a greater intensity (M = 8.11) than the samples that received neoadjuvant chemoradiation (M = 3.18) (Figure S1b). Furthermore, MUC16 expression was found in precancerous lesions of pancreatic cancer, intraductal papillary mucinous neoplasms (IPMN), and pancreatic intraepithelial neoplasms (PanIN). In the NCT group, four specimens contained IPMN tissue, with three samples staining for MUC16 expression (M = 2.75). In the non-NCT tumor specimens, varying levels of MUC16 expression were found in IPMN (*N* = 1; M = 2), PanIN (*N* = 2; M = 0.5), and pancreatitis (*N* = 1; M = 0) structures.

A summary of MUC16 expression characteristics and correlation in the sample population can be found in Supplementary Tables S1 and S2. Representative images show the range of MUC16 expression in each group (Figure S2).

### 3.3. MUC16 Retention after Chemotherapy Treatment

**Figure 2:**
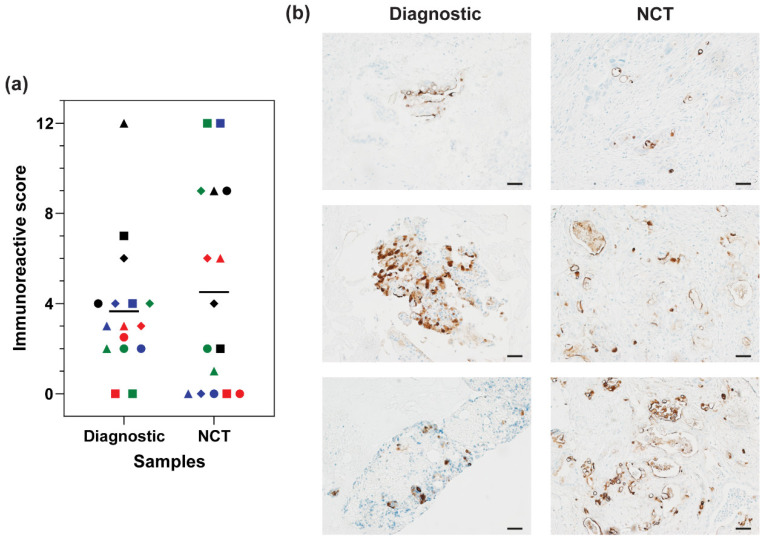
MUC16 retention in PDAC patient samples after chemotherapy treatment. (**a**) Distribution of MUC16 stain intensity in matched diagnostic and NCT samples. Matched samples are indicated by the same color and shape. (**b**) Representative images of the distribution of MUC16 stain intensity in the matched diagnostic and NCT samples. Scale bar = 50 µm.

To determine the impact of chemotherapy treatment on MUC16 expression in patient samples, MUC16 expression was compared between the matched diagnostic and NCT samples. MUC16 expression was retained after NCT in patient samples (Figure 2). Expression was comparable after chemotherapy treatment between the matched diagnostic and NCT samples (Figure 2a). MUC16 expression increased in 6 samples, decreased in 8 samples, and remained the same in 2 samples after the NCT regimen. In 4 samples, MUC16 expression was lost in the NCT sample compared to their matched diagnostic sample. However, the mean MUC16 stain intensity between the diagnostic and NCT groups was comparable (Diagnostic: M = 3.66; NCT: M = 4.5). No trend was observed in MUC16 expression after NCT in the patient samples.

### 3.4. MUC16 Expression in Matched Adjacent Tissue

**Figure 3:**
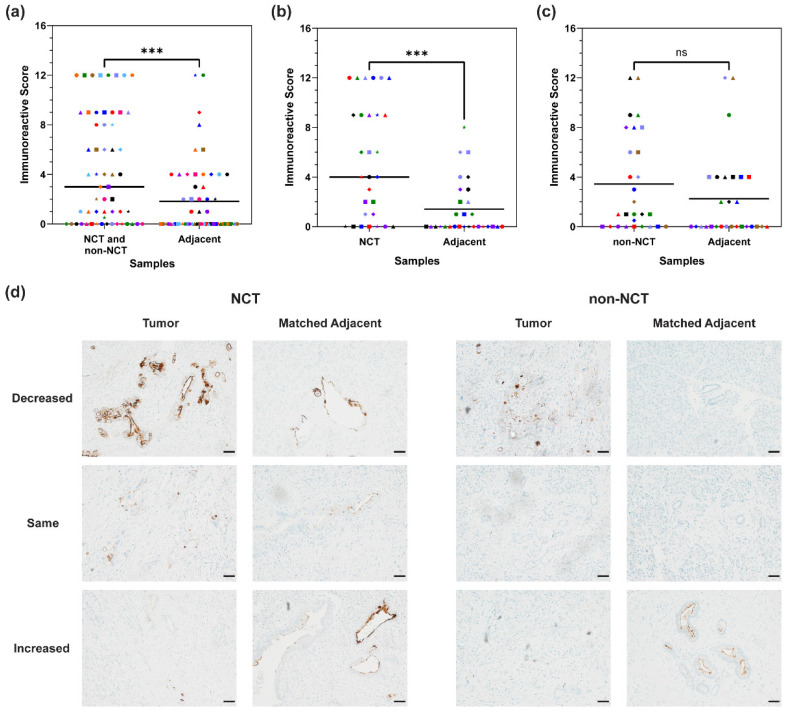
Differential MUC16 expression between PDAC and matched adjacent tissues. (**a**–**c**) MUC16 stain intensity in treated samples compared to matched adjacent tissue. The matched data points are indicated with the same color and shape. The mean line is plotted in the dot plot. Data were analyzed using the Wilcoxon matched pairs test. *p* < 0.05; *** 0.0001 < *p* < 0.001. Differential MUC16 expression in (**a**) all tumors and matched adjacent tissues. (**b**) NCT samples and matched adjacent tissues. (**c**) non-NCT samples and matched adjacent tissues. (**d**) Representative images of the differential MUC16 expression. Images taken at 20× magnification. Scale bar = 50 µm.

Clear margins are needed to distinguish the tumor from surrounding normal tissue for a successful tumor resection. MUC16 expression was significantly diminished in matched adjacent samples compared to the NCT or non-NCT matched samples (*p* = 0.0002) (Figure 3a). In matched adjacent samples with only benign or benign and precancerous tissue, expression decreased in 22 samples (61.1%) (NCT and non-NCT: M = 6.39; matched adjacent: M = 0.5), increased in 3 samples (8.3%) (NCT and non-NCT: M = 1; matched adjacent: M = 6.3), and stayed the same in 11 samples (30.6%) (M = 0) compared to the matched NCT or non-NCT sample. In all, 31 matched adjacent tissues showed a decrease in MUC16 expression (54.4%) (NCT and non-NCT: M = 7.02; matched adjacent: M = 1), and 10 increased in MUC16 expression (17.5%) (NCT and non-NCT: M = 1.5; matched adjacent: M = 5.3) compared to the matched NCT or non-NCT primary tumor tissue. Sixteen adjacent tissues had the same MUC16 expression intensity as their matched NCT or non-NCT tissue (28.1%) (M = 1.25). Out of these 16 samples, 13 had no MUC16 expression (11 benign and 2 cancerous), while 3 expressed MUC16 (2 tumors and 1 tumor and benign tissue). In the NCT specimens, there was a statistically significant change in MUC16 expression between the NCT tissues and the matched adjacent tissues (*p* = 0.0003) (Figure 3b). A total of 17 (58.6%) adjacent samples matched with NCT samples weakly expressed MUC16 (NCT: M = 8.24; matched adjacent: M = 0.88), 8 had the same MUC16 expression (27.6%) (M = 1), and 4 had an increased MUC16 expression (13.8%) (NCT: M = 2; matched adjacent: M = 4.5). In only benign or benign and precancerous tissue, 12 samples decreased in MUC16 expression (63.16%), 1 sample had increased MUC16 expression (5.26%), and 6 samples had the same MUC16 expression (31.58%) compared to the matched NCT tissue. There was not a statistically significant difference in MUC16 expression between the non-NCT samples and matched adjacent tissue (*p* = 0.1446) (Figure 3c). Fourteen non-NCT matched adjacent samples decreased in MUC16 expression (50%) (non-NCT: M = 5.54; matched adjacent: M = 1.14), 6 increased in MUC16 expression (21.4%) (non-NCT: M = 1.17; matched adjacent: M = 5.83), and 8 had the same MUC16 expression (28.6%) (M = 1.5). Furthermore, in matched adjacent samples with only benign for benign and precancerous tissue, 10 samples decreased in MUC16 expression (58.8%), 2 samples increased in MUC16 expression (11.8%), and 5 samples had the same MUC16 expression (29.4%). Of note, positive lymph node status in the patient was associated with tumor present in the matched adjacent tissues (Supplementary Table S3). Representative images showing the differing expression patterns in NCT or non-NCT specimens and the matched adjacent tissues can be found in Figure 3d.

## 4. Discussion

Previous studies in our lab have reported targeting MUC16 in PDAC preclinical models using an NIR-fluorescent antibody–dye conjugate [5,20]. Therefore, we investigated MUC16 expression after NCT in patient samples to reflect tumor heterogeneity and clinical treatments. We observed the presence of MUC16 expression in chemotherapy and chemoradiation-treated PDAC patient samples. Additionally, there was minimal change in MUC16 expression between the matched diagnostic and NCT samples. This indicates that MUC16-targeted therapies may have potential use after NCT in PDAC patients; however, a broader study will be needed to confirm these results.

One important point of consideration is the expression of MUC16 in neoadjuvant chemoradiation-treated tissues. There appears to be a trend of lower MUC16 expression in samples that underwent neoadjuvant chemoradiation therapy when compared to samples that underwent NCT. To confirm these results, it will be necessary to study the expression of MUC16 in a broader chemoradiation treatment group.

Consensus in the literature demonstrates that MUC16 is expressed in 60–80% of PDAC tissues, with minimal MUC16 expression in normal pancreatic tissues [20,32,33]. Compared to the NCT group, weaker MUC16 expression was found in the non-NCT samples. According to the IRS expression classification, only 48.4% of non-NCT samples expressed MUC16; however, some of the samples that were classified as non-expressing weakly expressed MUC16 (IRS of 0.5 or 1). Overall, 67.7% (21/31) of samples expressed MUC16 to varying degrees. Although there was not a significant difference in the expression of MUC16 between the non-NCT samples and the normal pancreas, a majority of non-NCT samples (67.7%) did express MUC16. This pattern of expression supported the established trend of MUC16 expression in PDAC tissues. The weaker expression in the non-NCT samples compared to the NCT samples may be due to heterogeneous protein expression, most likely leading to the lack of a statistically significant difference between MUC16 expression in non-NCT and normal pancreatic samples. The trend of weaker expression may also be due to the tumor stage at diagnosis. Previous research has indicated that MUC16 expression may increase as PDAC progresses [32]. Therefore, more advanced tumors may have higher MUC16 expression. Since the NCT tumors were restaged after chemotherapy, these patients most likely had a more advanced disease prior to the neoadjuvant therapy regimen compared to the non-NCT samples. Finally, the normal pancreatic tissue specimens expressed MUC16 at greater levels than expected, potentially caused by background staining. Mucin primary antibodies are known to have the potential for nonspecific background staining [43,44]. A majority of the normal pancreatic specimens weakly expressed MUC16 in the cytoplasm (85.7%), excluding the tissues that did not express MUC16. However, most non-NCT and NCT specimens displayed apical staining (NCT: 77.1%; non-NCT: 67.7%). Since most antibody binding occurred in the normal pancreatic cytoplasm, this may have been nonspecific staining. In all, the lack of significant difference between the non-NCT and normal pancreatic specimens may be due to the early tumor stage, small sample size, heterogeneous protein expression, and nonspecific staining.

In addition, the ability to detect tumor margins was investigated for future applications of FGS. In our samples, the surrounding benign or precancerous tissue matched to the NCT or non-NCT samples minimally expressed MUC16, indicating that high tumor-to-background ratios and tissue differentiation may be achievable in PDAC. However, MUC16 was still expressed in some of the matched adjacent samples. Of those matched adjacent tissues expressing MUC16, 15 out of 24 samples (62.5%) contained MUC16 expression in infiltrating tumor cells. Additionally, tumor foci were found in other matched adjacent samples (22; NCT: 10, non-NCT: 11). A majority of these tumor foci expressed MUC16, showing the potential ability to detect these residual tumor sections using intraoperative tools (NCT: 8/10; non-NCT: 8/11). While pancreatic cancer has a high desmoplastic nature, previous research has shown the ability of antibody–dye conjugates to target the tumor in clinical trials [21,25,45]. Therefore, an FGS conjugate may be able to target those infiltrating tumor cells to increase the possibility of a complete resection. In addition to tumor foci, some benign ducts and ampulla regions expressed MUC16 in the matched adjacent tissue (NCT: 13.3%, M = 2.75; non-NCT: 17.9%, M = 3.8). Finally, minimal precancerous lesions were present in the matched adjacent tissue sections. The NCT adjacent tissue samples had three samples with PanIN structure, but only one was positive for MUC16 expression. Likewise, the non-NCT matched adjacent samples had five samples with PanIN tissue, with one moderately expressing MUC16.

One potential limitation of this study is the sample size. In the future, a large-scale study of MUC16 expression after NCT and chemoradiation in PDAC patients will be needed to confirm these results. Another potential limitation is the inclusion criteria for the NCT samples. Since these samples were from patients deemed eligible for surgery, we potentially lost a cohort of patients who received NCT but did not have a distinct tumor reduction. Therefore, our results for MUC16 expression may have been affected by the loss of NCT patient samples that did not undergo surgical resection. Additionally, many other proteins are upregulated in PDAC along with MUC16 [17,46,47]. This study is potentially limited by the inclusion of only one protein. To continue to grow and understand this field, the effect of NCT will need to be studied on other proteins. Finally, protein expression has the potential to fluctuate between tissue slices. Therefore, future research will need to take this into account.

## 5. Conclusions

In recent decades, NCT has increased in clinical use to treat pancreatic cancer [2,12]. These data indicate that MUC16 is still expressed in PDAC tissues after chemotherapy treatment. In all, these data indicate the need for future research on the expression of MUC16 after neoadjuvant therapy.

## Figures and Tables

**Table 1:** Tissue sample characteristics.

	No Prior Neoadjuvant Chemotherapy Treatment (Non-NCT) (%) (*N* = 31)	Neoadjuvant Chemotherapy Treatment (NCT) (%) (*N* = 35)
Tumor grade		
Well Differentiated	6 (19.4%)	9 (25.7%)
Moderately Differentiated	15 (48.4%)	16 (45.7%)
Poorly Differentiated	10 (32.3%)	10 (28.6%)
Primary tumor designation		
T1	5 (16.1%)	11 (31.4%)
T2	11 (35.5%)	20 (57.1%)
T3	14 (45.2%)	3 (8.6%)
T4	1 (3.2%)	1 (2.9%)
Lymph node designation		
N0	7 (22.6%)	15 (42.9%)
N1	18 (58.1%)	16 (45.7%)
N2	6 (19.4%)	4 (11.4%)
Metastatic disease designation		
MX	4 (12.9%)	7 (20%)
M0	26 (83.9%)	28 (80%)
M1	1 (3.2%)	0 (0%)
AJCC Stage		
IA	3 (9.7%)	6 (17.1%)
IB	2 (6.5%)	10 (28.6%)
IIA	11 (35.5%)	14 (40%)
IIB	10 (32.3%)	4 (11.4%)
IIIA	3 (9.7%)	0 (0%)
IIIB	1 (3.2%)	1 (2.9%)
IV	1 (3.2%)	0 (0%)
Fibrosis classification		
All tumor	1 (3.2%)	1 (2.9%)
More tumor than fibrosis	20 (64.5%)	9 (25.7%)
More fibrosis than tumor	10 (32.3%)	25 (71.4%)
All fibrosis	0 (0%)	0 (0%)
Neoadjuvant therapy treatment		
FOLFIRINOX-based	NA	14 (40%)
FOLFIRINOX + radiation	NA	17 (48.6%)
Gemcitabine-based	NA	4 (11.4%)
Grade of pathological response		
No response	NA	1 (2.86%)
Minimal response	NA	14 (40%)
Moderate response	NA	17 (48.57%)
Marked response	NA	3 (8.57%)

## Data Availability

Data will be made available upon request to the corresponding author.

## Supplementary Materials

The following supporting information can be downloaded at: https://www.mdpi.com/article/10.3390/cancers16203439/s1, Table S1: MUC16 expression characteristics. Table S2: MUC16 expression intensity trends based on tumor characteristics. Table S3: Association of tumor presence in matched adjacent samples with primary tumor clinical characteristics. Figure S1: Distribution of MUC16 expression in the neoadjuvant therapy regimens. Figure S2: Representative distribution of MUC16 expression intensity in NCT and non-NCT samples.
