# Supplementary material for: MUC16 Retention after Neoadjuvant Chemotherapy in Pancreatic Ductal Adenocarcinoma
Source: Cancers (Basel). 2024 Oct 10;16(20):3439. doi: 10.3390/cancers16203439 (PMC11506185; doi:10.3390/cancers16203439)
Supplement: Supplementary file 1 [file cancers-16-03439-s001.zip › cancers-3192459-supplementary.pdf]

Supplementary Material

1. Results

1.2. MUC16 Expression Characteristics

The non-NCT and NCT groups showed a similar trend in sample distribution particularly in the categories: trace, 1+, and 2+. The negative expression (non-NCT: 32.3%, NCT: 20.0%) and 3+ expression (non-NCT: 25.8%, NCT: 45.7%) categories showed the largest difference in sample distribution. In addition, there was no distinct trend difference in the location of MUC16 expression in the non-NCT and NCT groups. A majority of samples showed an apical staining pattern in both groups. Conversely, the normal pancreas samples had a different trend in MUC16 expression location compared to the non-NCT and NCT groups as a majority of samples displayed cytoplasmic expression. Additionally, many of the samples with cytoplasmic stain also weakly expressed MUC16 (75% cytoplasmic compared to 25% apical weak expression). This weak cytoplasmic stain may be indicative of non-specific background stain. Finally, the percentage of positive cells and IRS classification had the most distinct trend differences between the non-NCT and NCT groups. A majority of NCT samples classified with 25% of tumor cells or more staining positive for MUC16 expression compared to the non-NCT samples (non-NCT: 38.7%, NCT: 71.4%). In the IRS classification, the non-NCT group had a higher portion of samples classified with no expression (non-NCT: 51.6%, NCT: 25.7%), while the NCT group contained a higher portion of samples classifying with medium and high expression (non-NCT: 38.7%, NCT: 65.7%). These trends are shown in **Supplementary Table S1.**

2. Figures and Tables

Supplementary Table S1. MUC16 expression characteristics.

	Non-NCT (%) (*N* = 31)	NCT (%) (*N* = 35)	Normal Tissue (%) (*N* = 10)
**Expression intensity**			
**Negative**	10 (32.3%)	7 (20%)	3 (30%)
**Trace**	1 (3.2%)	0 (0%)	2 (20%)
**1+**	5 (16.1%)	4 (11.4%)	4 (40%)
**2+**	7 (22.6%)	8 (22.9%)	0 (0%)
**3+**	8 (25.8%)	16 (45.7%)	1 (10%)
**Percentage of positive cells**			
**0%**	10 (32.3%)	7 (20%)	3 (30%)
**Less than 25%**	9 (29%)	3 (8.6%)	2 (20%)
**25-50%**	3 (9.7%)	9 (25.7%)	3 (30%)
**51-75%**	3 (9.7%)	7 (20%)	2 (20%)
**Greater than 75%**	6 (19.4%)	9 (25.7%)	0 (0%)
**Location of expression**			
**Apical**	18 (58.1%)	19 (54.29%)	1 (10%)
**Membrane**	0 (0%)	1 (2.86%)	0 (0%)
**Cytoplasmic**	0 (0%)	0 (0%)	5 (50%)
**Apical and membrane**	0 (0%)	2 (5.71%)	0 (0%)
**Apical and cytoplasmic**	1 (3.2%)	1 (2.86%)	1 (10%)
**Apical and secretion**	0 (0%)	3 (8.57%)	0 (0%)
**Apical, membrane, and secretion**	1 (3.2%)	2 (5.71%)	0 (0%)
**Apical, membrane, islet, and secretion**	1 (3.2%)	0 (0%)	0 (0%)
**No stain**	10 (32.3%)	7 (20%)	3 (30%)
**Immunoreactive score classification**			
**No expression (0-1)**	16 (51.6%)	9 (25.7%)	6 (60%)
**Mild expression (2-3)**	3 (9.7%)	3 (8.6%)	4 (40%)
**Medium expression (4-8)**	7 (22.6%)	11 (31.4%)	0 (0%)
**High expression (9-12)**	5 (16.1%)	12 (34.3%)	0 (0%)

Supplementary Table S2. MUC16 expression intensity trends based on tumor characteristics.

	**Non-NCT (*N* = 31)**	**NCT (*N*=35)**
	Mean IRS (% of n)	Mean IRS (% of n)
**Tumor Grade**		
**Well differentiated**	3.67 (19.3%)	6.89 (25.7%)
**Moderately differentiated**	1.97 (48.4%)	5.13 (45.7%)
**Poorly differentiated**	6 (32.3%)	5.6 (28.6%)
**Primary tumor designation**		
**T1**	2.2 (16.1%)	4.91 (31.4%)
**T2**	2.73 (35.5%)	5.95 (57.1%)
**T3**	4.18 (45.2%)	8.67 (8.6%)
**T4**	12 (3.2%)	1 (2.9%)
**Lymph node designation**		
**N0**	2.21 (22.6%)	4.33 (42.9%)
**N1**	4.72 (58.1%)	6.69 (45.7%)
**N2**	1.83 (19.3%)	7 (11.4%)
**Metastatic disease designation**		
**MX**	6 (12.9%)	8 (20%)
**M0**	3.37 (83.9%)	5.14 (80%)
**M1**	0 (3.2%)	-
**AJCC stage**		
**IA**	3 (9.677%)	3.83 (17.1%)
**IB**	4 (6.452%)	4.6 (28.6%)
**IIA**	2.23 (35.484%)	7.29 (40%)
**IIB**	4.7 (32.258%)	7 (11.4%)
**IIIA**	3.67 (9.677%)	-
**IIIB**	12 (3.226%)	1 (2.9%)
**IV**	0 (3.226%)	-
**Neoadjuvant Therapy Treatment**		
**FOLFIRINOX-based**	NA	7.71 (40%)
**FOLFIRINOX + radiation**	NA	3.18 (48.6%)
**Gemcitabine-based**	NA	9.5 (11.4%)
**Fibrosis classification**		
**All tumor**	0 (3.2%)	1 (2.9%)
**More tumor than fibrosis**	3.3 (64.5%)	7.78 (25.7%)
**More fibrosis than tumor**	4.55 (32.3%)	5.16 (71.4%)
**Grade of pathological response**		
**No response**	NA	12 (2.8%)
**Minimal response**	NA	5.93 (40%)
**Moderate response**	NA	5.12 (48.6%)
**Maximum response**	NA	9 (8.6%)

Supplementary Table S3. Association of tumor presence in matched adjacent samples with primary tumor clinical characteristics.

	**Matched adjacent for Non-NCT (*N* = 28)**	**Matched Adjacent for NCT (*N* = 29)**
	Tumor Presence (% of n)	Tumor Presence (% of n)
**Tumor Grade**		
**Well differentiated**	3 (10.7%)	3 (10.3%)
**Moderately differentiated**	2 (7.1%)	6 (20.7%)
**Poorly differentiated**	5 (17.9%)	1 (3.4%)
**Primary tumor designation**		
**T1**	0 (0%)	3 (10.3%)
**T2**	4 (14.3%)	6 (20.7%)
**T3**	5 (17.9%)	1 (3.4%)
**T4**	1 (3.6%)	0 (0%)
**Lymph node designation**		
**N0**	1 (3.6%)	2 (6.9%)
**N1**	7 (25.0%)	7 (24.1%)
**N2**	2 (7.1%)	1 (3.4%)
**Metastatic disease designation**		
**MX**	1 (3.6%)	1 (3.4%)
**M0**	8 (28.6%)	9 (31.0%)
**M1**	1 (3.6%)	-
**AJCC stage**		
**IA**	0 (0%)	0 (0%)
**IB**	0 (0%)	4 (13.8%)
**IIA**	3 (10.7%)	5 (17.2%)
**IIB**	4 (14.3%)	1 (3.4%)
**IIIA**	1 (3.6%)	-
**IIIB**	1 (3.6%)	0 (0%)
**IV**	1 (3.6%)	-
**Fibrosis classification**		
**All tumor**	1 (3.6%)	0 (0%)
**More tumor than fibrosis**	6 (21.4%)	3 (10.3%)
**More fibrosis than tumor**	3 (10.7%)	7 (24.1%)

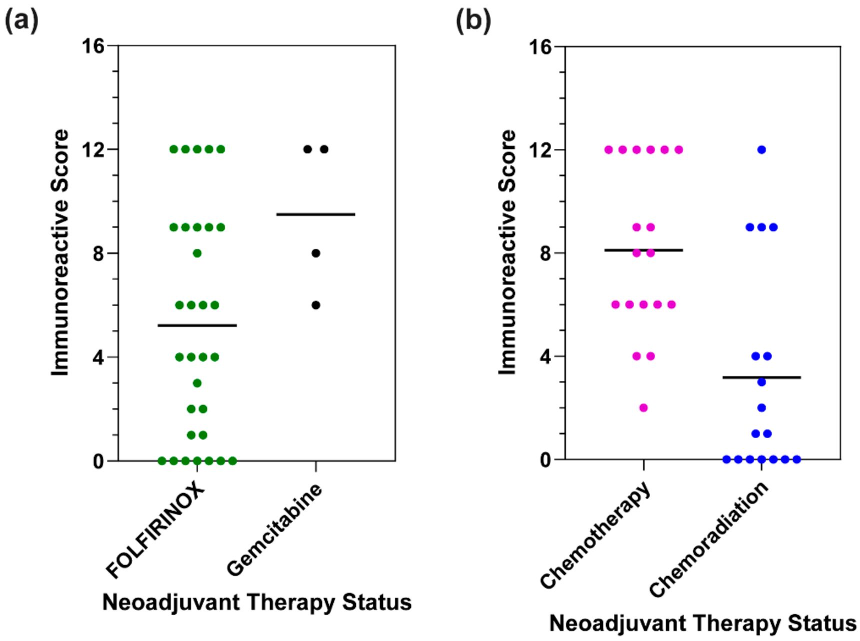

Supplementary Figure S1. Distribution of MUC16 expression in the neoadjuvant therapy regimens. (a,b) The individual data points are plotted with the mean indicated by the line. **(a)** Comparison of MUC16 expression after neoadjuvant chemotherapy regimens, FOLFIRINOX and gemcitabine. **(b)** Comparison of MUC16 expression between neoadjuvant chemotherapy and neoadjuvant chemoradiation treatments.

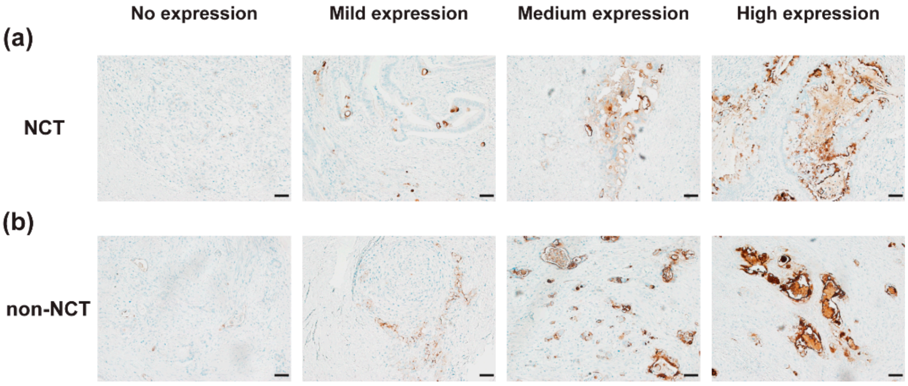

Supplementary Figure S2. Representative distribution of MUC16 expression intensity in NCT and non-NCT samples. IRS was classified according to 0-1 (no expression), 2-3 (mild expression), 4-8 (medium expression), and 9-12 (high expression). Images taken at 20x magnification. Scale bar = 50 μm. **(a).** Samples treated with neoadjuvant chemotherapy prior to surgical resection. **(b).** Samples that received no neoadjuvant chemotherapy treatment prior to surgical resection.
